# Effectiveness of diaphragmatic ultrasound as a predictor of successful weaning from mechanical ventilation: a systematic review and meta-analysis

**DOI:** 10.1186/s13054-023-04430-9

**Published:** 2023-05-05

**Authors:** Henry M. Parada-Gereda, Adriana L. Tibaduiza, Alejandro Rico-Mendoza, Daniel Molano-Franco, Victor H. Nieto, Wanderley A. Arias-Ortiz, Purificación Perez-Terán, Joan R. Masclans

**Affiliations:** 1 Intensive Care Unit Clínica Reina Sofia, Clínica Colsanitas, Grupo de Investigación en Nutrición Clínica y Rehabilitación, Fundación Universitaria Sanitas, Keralty Bogotá- Colombia, Universidad del Rosario Bogotá- Colombia, Faculty of Medicine. Research group Medicina Comunitaria y Salud Colectiva Universidad El Bosque, Bogotá, Colombia; 2 Physician Subinvestigator Oficina de Investigaciones Hospital San Ignacio, Bogotá, Colombia; 3 Epidemiology Master’s Degree Coordinator. Universidad El Bosque. Faculty of Medicine. Research group Medicina Comunitaria y Salud Colectiva. Bogotá Colombia, Universidad El Bosque, Bogotá, Colombia; 4 Intensive Care Cobos Medical Center – Universidad El Bosque, group Recerca-GRIBOS, Bogota, Colombia; 5 Head of Intensive Care Unit Centro de Tratamento e Investigación sobre Cáncer CTIC, group Recerca- GRIBOS, Bogotá, Colombia; 6 Master in Epidemiology, Specialist in Applied Statistics, Universidad El Bosque. Faculty of Medicine. Research group Medicina Comunitaria y Salud Colectiva, Bogotá, Colombia; 7 Critical Care Department Hospital del Mar-Parc de Salut MAR. GREPAC-Group Recerca Departamento de Medicina y Ciencias de la Vida Universitat Pompeu Fabra (UPF), Barcelona, Spain; 8 Director de Docencia PSMAR, Intensive Care Unit Hospital del Mar. Professor of Medicine Universitat Pompeu Fabra (UPF) IMIM (GREPAC - Group Recerca Patologia Critica) Departamento de Medicina Y Ciencias de la Vida (MELIS), Universidad Pompeu Fabra (UPF), Barcelona, Spain

**Keywords:** Diaphragm, Ultrasonography, Diagnostic imaging, Weaning, Mechanical ventilation, Airway extubation

## Abstract

**Background:**

Several measurements have been used to predict the success of weaning from mechanical ventilation; however, their efficacy varies in different studies. In recent years, diaphragmatic ultrasound has been used for this purpose. We conducted a systematic review and meta-analysis to evaluate the effectiveness of diaphragmatic ultrasound in predicting the success of weaning from mechanical ventilation.

**Methods:**

Two investigators independently searched PUBMED, TRIP, EMBASE, COCHRANE, SCIENCE DIRECT, and LILACS for articles published between January 2016 and July 2022. The methodological quality of the studies was assessed using the Quality Assessment of Diagnostic Accuracy Studies-2 tool; additionally, the certainty of the evidence is evaluated using the GRADE (Grading of Recommendations Assessment, Development, and Evaluation) methodology. Sensitivity and specificity analysis was performed for diaphragmatic excursion and diaphragmatic thickening fraction; positive and negative likelihood ratios and diagnostic odds ratios (DOR) with their confidence intervals (95% CI) were calculated by random effects analysis, summary receiver operating characteristic curve was estimated. Sources of heterogeneity were explored by subgroup analysis and bivariate meta-regression.

**Results:**

Twenty-six studies were included, of which 19 were included in the meta-analysis (1204 patients). For diaphragmatic excursion, sensitivity was 0.80 (95% CI 0.77–0.83), specificity 0.80 (95% CI 0.75–0.84), area under the summary receiver operating characteristic curve 0.87 and DOR 17.1 (95% CI 10.2–28.6). For the thickening fraction, sensitivity was 0.85 (95% CI 0.82–0.87), specificity 0.75 (95% CI 0.69–0.80), area under the summary receiver operating characteristic curve 0.87 and DOR 17.2 (95% CI 9.16–32.3). There was heterogeneity among the included studies. When performing a subgroup analysis and excluding studies with atypical cutoff values, sensitivity and specificity increased for diaphragmatic thickening fraction; sensitivity increased and specificity decreased for diaphragmatic excursion; when comparing studies using pressure support (PS) versus T-tube, there was no significant difference in sensitivity and specificity; bivariate meta-regression analysis shows that patient position at the time of testing was a factor of heterogeneity in the included studies.

**Conclusions:**

Measurement of diaphragmatic excursion and diaphragmatic thickening fraction predict the probability of successful weaning from mechanical ventilation with satisfactory diagnostic accuracy; however, significant heterogeneity was evident in the different included studies. Studies of high methodological quality in specific subgroups of patients in intensive care units are needed to evaluate the role of diaphragmatic ultrasound as a predictor of weaning from mechanical ventilation.

**Supplementary Information:**

The online version contains supplementary material available at 10.1186/s13054-023-04430-9.

## Background

The process of weaning from mechanical ventilation remains one of the most critical challenges in patients undergoing mechanical ventilation in the intensive care unit (ICU) [1]; the multidisciplinary team must study the optimal time for weaning from the mechanical ventilator as premature weaning may lead to weaning failure and thus increase the risk of hospital acquired infections, costs in care, ICU length of stay, hospital length of stay and diaphragmatic dysfunction [2, 3].

Current guidelines recommend several indices applied at the bedside to predict successful weaning from mechanical ventilation. However, they have yet to prove ideal [4], probably due to the heterogeneity of critically ill patients, which limits the predictive ability of these indices in different patient subgroups [5]. A spontaneous breathing trial (SBT) is an appropriate way to prepare the patient for extubation [6]; however, even after successful SBT, failure rates and subsequent reintubation can exceed 20% in the highest-risk patients [7].

Patients on mechanical ventilation may have a multifactorial deterioration of diaphragmatic function that can lead to weaning failure and prolongation of invasive mechanical ventilation [8, 9]; therefore, assessing diaphragmatic function could help predict the patient's ability to maintain spontaneous breathing over time [10].

The use of diaphragmatic ultrasound in the intensive care unit is a technique of growing interest due to its portability, speed, and safety. Its use allows reporting on the structural and functional status of the diaphragm and can predict the probability of successful mechanical ventilator weaning [11]. Although some studies have demonstrated the usefulness of ultrasound in predicting the success of mechanical ventilator weaning, others have shown controversial results that continue to motivate continued research of this technique. This systematic review and meta-analysis aim to compile the best available evidence to elucidate the effectiveness of diaphragmatic ultrasound as a predictor of successful weaning from the mechanical ventilation.

## Materials and methods

### Search for studies

A systematic review and meta-analysis of observational studies involving intubated patients connected to mechanical ventilation who underwent uni- or bilateral diaphragmatic ultrasound to assess diaphragm function prior to extubation was performed to identify whether there is an association between diaphragm function and extubation success. The systematic review protocol was registered in the Prospective International Register of Systematic Reviews (PROSPERO) CRD42022316349 database, and the systematic review was conducted according to the Preferred Reporting Items for Systematic Reviews and Meta-Analyses (PRISMA) guidelines.

Two investigators conducted independent systematic literature searches of PUBMED, TRIP, EMBASE, COCHRANE, SCIENCE DIRECT, and LILACS databases published between January 2016 through July 2022, discrepancies between the two investigators were resolved with the intervention of a third investigator. The terms diaphragm, diagnostic imaging, ultrasound, weaning, mechanical ventilation, extubation, ultrasonography, and articles in all languages were included.

Methodological quality assessment was performed independently by two investigators using the Quality Assessment of Diagnostic Accuracy Studies-2 (QUADAS-2) instrument, followed by the kappa coefficient to assess inter-investigator agreement; additionally, the certainty of the evidence is evaluated using the GRADE (Grading of Recommendations Assessment, Development, and Evaluation) methodology.

### Selection criteria and outcome measures

The meta-analysis included studies published between 2016 and 2022, and weaning success was defined as maintaining spontaneous breathing for the next 48 h after extubation.

### Inclusion criteria

Prospective or retrospective observational studies involving adult patients with more than 24 h of invasive mechanical ventilation in whom uni- or bilateral diaphragmatic ultrasound was performed during spontaneous breathing trial.

### Exclusion criteria

Non-primary studies, studies with less than 20 participants, patients with neuromuscular disease, studies in pregnant patients, case reports, animal studies, and editorials.

### Statistical analysis

Rev Man 5.4 the Cochrane Collaboration (2014) software was used for bias assessment, and Metadisc software (Hospital Ramón Y Cajal, Madrid, Spain) for meta-analysis [12]. Independent analyses were performed for diaphragm excursion (DE) and diaphragm thickening fraction (DTF); likewise, an independent analysis of the results according to hemi-diaphragm was assessed; weaning success was defined as the absence of disease in the 2 × 2 table. Sensitivity and specificity analysis was performed for the studies that evaluated DE and DTF, positive and negative likelihood ratios, and diagnostic odds ratios with their respective 95% confidence intervals (CI). A statistically significant value of P < 0.05 was considered.

Summary receiver operator characteristic curves (SROC) and area under the summary curve (AUSROC) were created to assess the accuracy of DE and DTF for predicting extubation success [13]. Publication bias was assessed using the funnel plot and Egger's statistic [14].

The Cochrane Q and I^2^ tests assessed heterogeneity, and the source of heterogeneity was assessed by meta-regression analysis and a subgroup analysis on both sensitivity and specificity. Study characteristics that could cause uncertainty related to the diagnostic accuracy of diaphragm ultrasound were examined; for example, the cutoff values used as a reference, the risk of bias assessed concerning index test and flow and times, the type of spontaneous breathing trial (pressure support (PS) or T-piece), the homogeneity versus heterogeneity of age, the prevalence of success, and the position of the patient. In addition, an analysis was performed to identify whether the cause of the requirement for mechanical ventilation could influence the diagnostic performance of ultrasound.

## Results

**Fig. 1:**
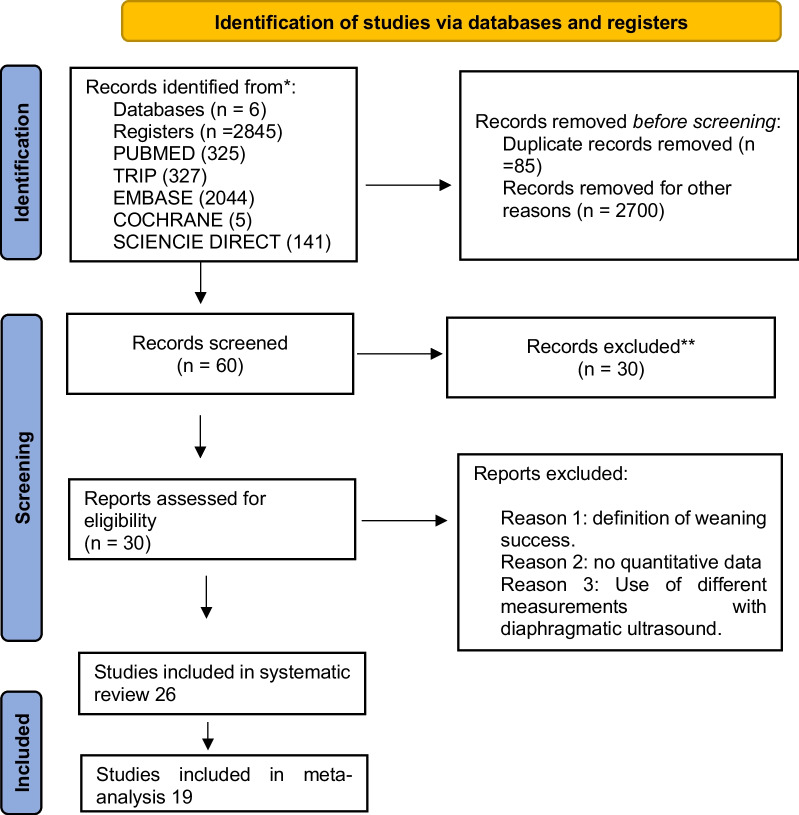
Study selection flowchart

In the initial search, 2845 articles were obtained in six databases, 85 duplicates and 2700 articles were eliminated by titles and abstracts, leaving 60 articles; subsequently, the full-text reading proceeded, eliminating 34 articles. (The detailed flow diagram is shown in Fig. 1.)

Twenty-six studies were included in the qualitative analysis of which 19 were included in the quantitative analysis. The meta-analysis included 1204 subjects, of whom 908 had DE assessment, and 945 had DTF assessment; in several studies, both DE and DTF were measured.

### Characteristics of the included studies

The different characteristics of the studies in the systematic review are listed in Table 1. Most of the studies were of the prospective cohort type, with the exception of [15], a retrospective observational study, and [16], a prospective randomized clinical trial.

**Table 1:** Studies included in the systematic review and meta-analysis

Autors	Country	Year	Design	n	Age	Reason for mechanical ventilation	Prevalence of success	Patient position during US	Cut-off success weaning	MV duration at inclusion (hours)	Evaluated Diaphragm	Mode/ sound	Tipe SBT	Weaning success definition
Jung B, et al	France	2016	Prospective cohort	40	58 (51–67)	ARF 65%	50%	20 a 30°	DTF ≥ 30%	> 48 h	Left and right	Not described	PS /T-tube	No reintubation or tracheostomy > 48 h after extubation
Blumhof et al	USA	2016	Prospective cohort	56	62 ± 17	ARF 73%	86.5%	Semi-recumbent 20–40°	DTF > 20%	> 24 h	Right	Mode B 7,5–10 MHz	PS	Spontaneous respiration > 48 h after extubation
Mariani Lf et al	France	2016	Prospective cohort	34	646 ± 14.8	Multicausal COPD 26%	100%	Semi-recumbent 30°	DE left _ > 11 mm ED right ≥ 10 mm	MV > 7 days	Left and right	3–5 MHz/ Mode M	T-tube	Spontaneous respiration > 72 h after extubation
Flevari et al	Greece	2016	Prospective cohort	27	65	Multicausal	Not described	Supine	DE right > 10 mm > 7 mm left	Not described	Left and right	5 MHz Mode B/ M	T-tube	SBT success, no reintubation or NIV > 48 h after extubation
Hayat et al	Pakistan	2017	Prospective observational	100	40.5	Respiratory disease 73%	76%	Supine	DE > 1.2 cm	Not described	Left and right	3.5 MHz/not described	Not described	Success in the next 48 h without the need for NIV or reintubation
Huang et al	China	2017	Prospective observational	40	84.25 ± 7.07	Multicausal COPD 52.5%	30%	Semi-incorporated	DE > 10 mm	> 48 h	Left and right	1.5 MHZ/ Mode M	T-tube	Unassisted respiration > 48 h
Farghaly et al	Egypt	2017	Prospective observational	54	Success group 65 (55–67.8) Failure group 62.5 (55–70.7)	Respiratory disease unspecified	74%	Semi-recumbent	DE ≥ 10.5 mm, Tdi ≥ 21 mm end inspiration, ≥ 10.5 mm Tdi end expiration, DTF ≥ 34.2%	Not described	Right	3.5 MHZ/Mode B	PS	Spontaneous respiration > 48 h after extubation
Osman et al	Egypt	2017	Prospective observational	68	56 (45–65)	Major surgery	73.5%	Semi-recumbent	DE > 10 mm DTF > 28%	Not described	Left and right	3.5 y 9–11 MHZ Mode M	T-tube	Spontaneous respiration > 48 h after extubation
Dres et al	France	2018	Prospective cohort	76	58 (48–68)	Multicausal ARF 40%	43.5%	Semi-incorporated	Contraction pressure > 7.2 cmH2O, DTF 25.8%	> 24 h	Left and right	4–12/Mode M	PS	Spontaneous respiration > 48 h after extubation
Yoo et al	Corea	2018	Prospective observational	60	69.5 (57.5–76)	Multicausal COPD 23.3%	78.3%	Not described	DE ≥ 1 cm and Δtdi ≥ 20%	> 48 h	Right	2–5; 6–13 /Mode M/ B	PS /T-tube	Success > 48 h without requiring NIV
Pirompanich et al	Thailand	2018	Prospective cohort	34	66.5 (± 13.5)	Multicausal COPD/ asthma 5.9%	73.5%	Semi-incorporated	DTF right ≥ 26%	> 24 h	Left and right	Lineal 10 MHZ/mode B/ M	T-tube	Spontaneous respiration > 48 h after extubation
Palkar et al	Norway	2018	Prospective cohort	73	71	Multicausal COPD/ asthma 21.9%	72.6%	Semi-recumbent 20–40°	Excursion-time index > 3.8%	> 24 h	Right	3.5 MHZ/Mode M	PS	Spontaneous respiration > 48 h after extubation
Tenza Lozano	Spain	2018	Prospective cohort	69	66 (53–78)	Multicausal respiratory disease 34.8%	63.7%	Semi-recumbent 20 a 40°	DTF > 24%	> 24 h	Right	Lineal 7 a 10 MHZ/ mode B/ M	PS/T-tube	Spontaneous respiration > 48 h after extubation
Theerawit et al	Thailand	2018	Prospective cross-sectional	62	66.48 (± 16,7)	Multicausal respiratory disease 19.3%	82.2%	Supine	DE > 12.85 mm	> 48 h	Left and right	1–5 MHZ/Mode B/ M	PS/T-tube	Spontaneous respiration > 48 h after extubation
Mowafy et al	Egypt	2019	Prospective randomized clinical trial	106	Group I 35.83(± 9.46) Group II 35.77(± 9.56)	Polytrauma	68.9%	Semi-recumbent 30 a 40°	DRSBI > 1.6	> 48 h	Right	2–5 MHZ Mode B/ M	PS	Spontaneous respiration > 48 h after extubation
Vivier et al	France	2019	Prospective multicenter	191	68 (±)	Respiratory disease 40%	83%	Not described	DE > 10 mm and DTF > 30%	vm > 7 days	Left and right	Lineal 4 y 10 MHZ Mode M	T-tube	Reintubation failure or death within 7 days after extubation
Varon et al	Colombia	2019	Prospective observational	84	58(35–51)	HRF 25%	79.8%	Semi-incorporated 45°	DC velocity > 2.9 cm/s in the SG; > 2.02 cm/s in the FG	> 48 h	Right	1–5 y 6–13 MHZ Mode M	PS/T-tube	Spontaneous respiration > 48 h after extubation
Soliman et al	Egypt	2019	Prospective observational	100	57.1 (± 14.5)	ARF unspecified 62%	80%	Not described	DTF > 29.5%	> 24 h	Left and right	Lineal 10 MHZ/Mode B/ M	PS	Success of SBT, no reintubation or death > 48 h
Eltrabili et al	Egypt	2019	Prospective observational	30	Success 52.7 (± 13.4) Failure 51.4 (± 13.1)	Abdominal sepsis	56.6%	Semi-recumbent	DTF > 30.7% and DE > 10.4 mm	> 48 h	Left and right	7.5-10MHZ Mode B/ M	PS	Successful spontaneous breathing and no use of NIV > 48 h after extubation
Abdelwahed et al	Egypt	2019	Prospective observational	65	Success 43.49 ± 12.88 Failure 40.85 ± 14.28	Multicausal respiratory disease 16.98%	78.4%	Semi-incorporated 30–45°	DTF > 30%	Not described	Left and right	10–15 MHZ Mode B/ M	T-tube	Spontaneous respiration > 48 h after extubation
Elshazly et al	Egypt	2020	Prospective observational	62	Success 65 (55–70) Failure 60.5 (52.2–70)	Respiratory disease COPD 24%	54.8%	Semi-incorporated	DE > 1.25 cm and DTF > 21.5%	> 24 h	Left and right	3.5 y 7–12 MHZ Mode B/ M	Not described	Spontaneous respiration > 48 h after extubation
Fossat et al	France	2021	Single-center prospective observational	100	66(± 15)	Multicausal COPD 14%	91%	Not described	RSBI and RSBI/DE had a value of 0.50 either at minute 5 and 0.55 at minute 25 of SBT to predict success	> 24 h	Right	5MHZ/Mode M	PS	Spontaneous respiration > 72 h after extubation
Shigang Li et al	China	2021	Prospective observational	101	> 65	Multicausal COPD 10%	68.3%	Not described	DTF ≥ 30%, DE ≥ 1.3 cm	> 24 h	Left and right	2–5, 5–13 MHZ/Mode B/ M	T-tube	No reintubation, NIV or tracheostomy > 48 h after extubation
Funda Gok et al	Turkey	2021	Prospective observational	62	57.6 (± 14.1)	Polytrauma 77%	64%	Not described	DTF > 27.5% DE > 1.3 cm	> 48 h	Right	2–4 MHZ/Mode M	T-tube	SBT success, no reintubation, no NIV > 48 h post-extubation
Alam M et al	Bangladesh	2022	Prospective observational	31	42 (± 16)	Multicausal COPD 32%	58%	Semi-recumbent 20–40°	DE ≥ 11.43 mm DTF 19.77%	> 11 days	Right	2–4-13 MHZ/Mode B/ M	T-tube	Spontaneous respiration > 48 h after extubation
Mawla et al	Egypt	2022	Prospective cohort	90	18–97	Respiratory disease COPD 36.7%	56.6%	Semi-recumbent	DE 1.3 cm DTF 13.5%, contraction velocity > 0.95 cm/s and relaxation rate 0.7 cm/sg	> 48 h	Right	Mode B 9 MHZ/M 4MHZ	PS	Spontaneous respiration > 48 h after extubation

From: Effectiveness of diaphragmatic ultrasound as a predictor of successful weaning from mechanical ventilation: a systematic review and meta-analysis

*DRSBI* rapid and shallow diaphragmatic breathing rate, *DE* diaphragmatic excursion, *DTF* diaphragm thickening fraction, *VM* mechanical ventilation, *PS* pressure support, *SBT* spontaneous breathing trial, *NIV* noninvasive ventilation, *US* ultrasound, *SG* success group, *FG* failure group, *MHZ* megahertz, *TDi* diaphragmatic thickness, *DC* Diaphragmatic contraction, *ARF* Acute respiratory failure, *COPD* chronic obstructive pulmonary disease, *HRF* hypoxemic respiratory failure

Those studies published between 2016 and 2022 were conducted in different countries such as eight from Egypt [1, 2, 16–21]; one from the USA [22]; five from France [10, 23–26]; one from Greece [27]; one from Pakistan [28]; two from China [29, 30]; one from Korea [15]; two from Thailand [31, 32]; one from Spain [33]; one from Norway [34]; one from Colombia [35]; one from Turkey [36]; one from Bangladesh [37].

Twelve studies evaluated DTF and DE [1, 2, 10, 15, 17, 20, 21, 26, 30, 35-37]; five studies evaluated only DE [24, 27–29, 32]; six studies evaluated only DTF [18, 19, 22, 23, 31, 33]; one study measured index excursion time [34]; two studies measured diaphragmatic rapid shallow breathing index (DRSBI) [10, 16]; and two studies measured contraction velocity [21, 35]. Most studies evaluated both diaphragms (57.7%), and 11 studies evaluated only the right diaphragm (42%).

Ultrasound measurements were performed in different positions, the most prevalent being semi-sitting from 20 to 45° reported in 16 studies [1, 2, 16, 17, 19–24, 24, 25, 31, 33, 35, 37]; three studies performed the measurement in a supine position [27, 28, 32], and six studies did not report the patient position [10, 15, 18, 26, 30, 36].

There was a variation of 24 to 48 h in the minimum duration of mechanical ventilation prior to inclusion in the studies; five studies did not describe the time on mechanical ventilation prior to the start of weaning; in one study, the duration of mechanical ventilation prior to inclusion was 11 days [37]; in two studies, it was seven days [24, 26]. Exclusion criteria for most studies were conditions affecting diaphragm function, predominantly phrenic nerve injury.

Different definitions of weaning were identified. Successful weaning was defined as the patient's ability to maintain spontaneous breathing within 48 h after extubation without requiring invasive or noninvasive ventilatory support or performance of tracheostomy. Failure of extubation was defined in most studies as the inability to maintain spontaneous breathing within 48 h after extubation with a requirement of noninvasive ventilation, high-flow nasal cannula, reintubation, or tracheostomy.

Regarding the weaning protocol, in all studies, patients were prepared by a spontaneous breathing trial, which was performed with pressure support (PS inspiratory pressure = 5–8 cmH2O and PEEP = 0–5 cmH2O) [1, 2, 10, 16, 18, 22, 25, 34], or using the T-tube [17, 19, 20, 24, 26, 30, 36, 37], some studies combined PS and T-tube [15, 23, 27, 29, 32, 33, 35], and two studies did not record the spontaneous breathing trial method [20, 28].

### Evaluation of methodological quality

**Fig. 2:**
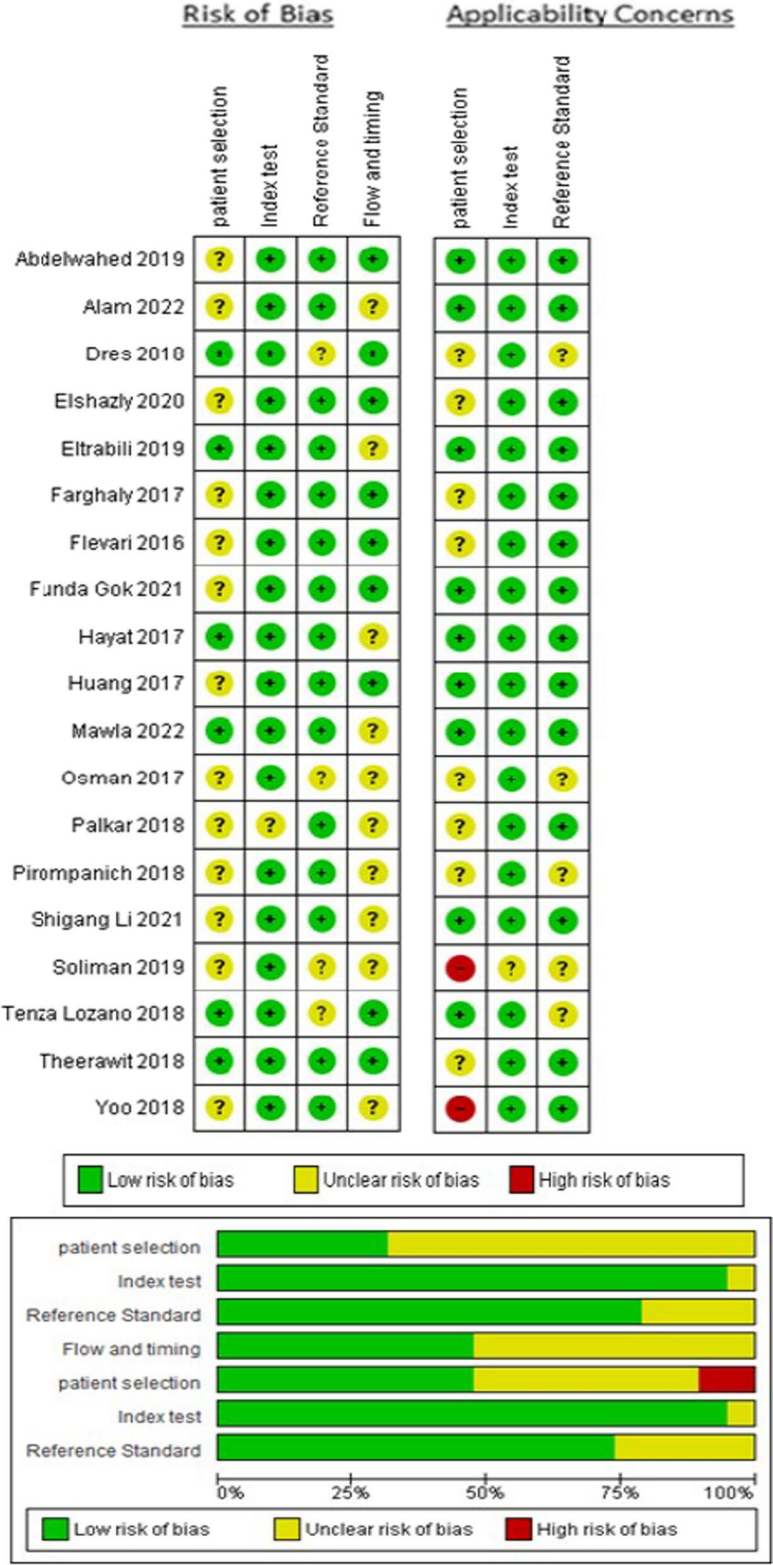
Risk of bias and applicability issues with Quality Assessment of Diagnostic Accuracy Studies (QUADAS)

The results of the risk of bias assessment for the studies included in the meta-analysis are shown in Fig. 2. There was agreement among the authors Cohen's Kappa 0.67 (95% CI 0.54–0.73); in 68% of the studies, there was the unclear risk of bias regarding patient selection, in 5.2% of the studies, there is an unclear risk of bias regarding the index test, in 21% of the studies, there is an unclear risk of bias regarding the standard reference, and 53% in flow and timing; regarding applicability concerns in two studies (10.5%), there was severe concern regarding patient selection [15, 18] unclear concern in one (5.2%) study regarding the index test [18] and in five (26.3%) studies regarding the standard reference [17, 18, 25, 31, 33]. The evaluation of the certainty of the articles included in the meta-analysis shows a moderate accuracy of the test (diaphragmatic ultrasound) (see Additional file 1: Table S1).

In most studies, diaphragmatic ultrasonography was performed during the spontaneous breathing trial with a variation of the exact timing of the test, and in other studies, diaphragmatic ultrasonography was performed before and after extubation. Most studies did not clearly report the elapsed time between diaphragmatic ultrasound and extubation [2, 17–21, 25, 27, 28, 33, 34].

The method of patient selection was not reported in most studies. Several studies did not present the flowchart explaining in detail the patient selection and follow-up [1, 15, 17, 18, 21, 28, 30, 31, 35, 37].

The outcome of weaning varied according to the definition of extubation success or failure and, in some studies, needed to be clearly defined. In most of the included studies, the index test (ultrasound of the diaphragm) was interpreted without knowing the outcome of weaning.

**Fig. 3:**
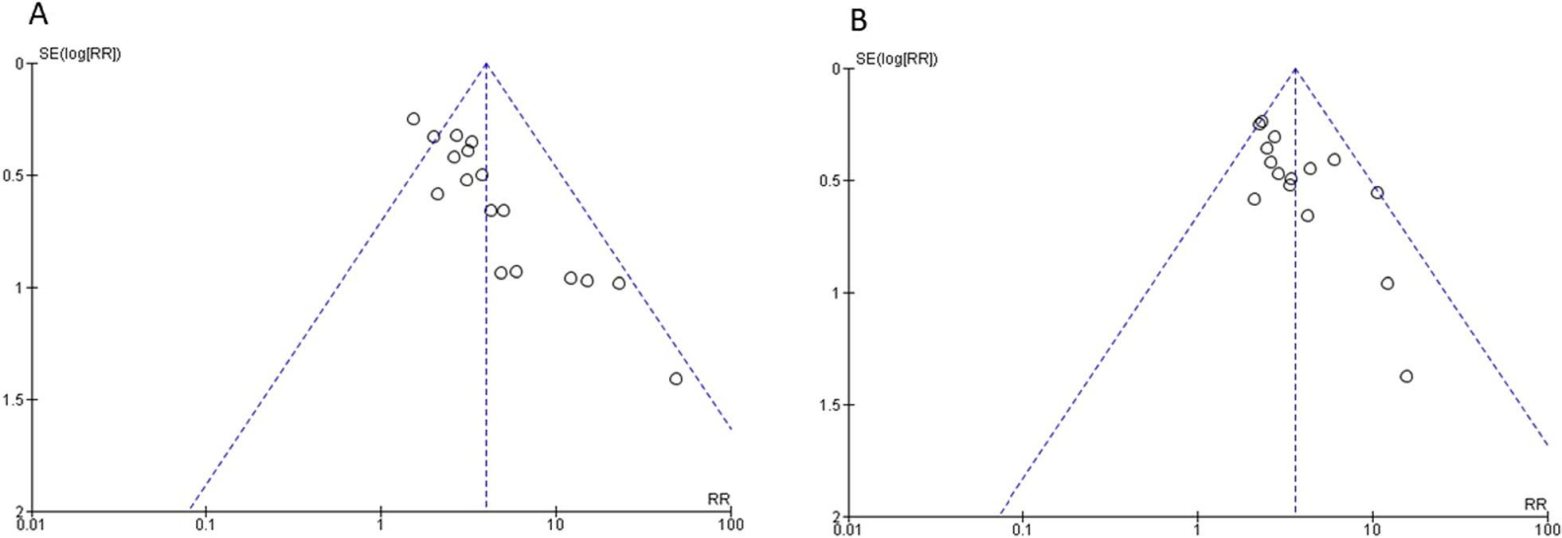
Funnel plot for diaphragmatic excursion (**A**) and diaphragm thickening fraction (**B**)

No publication bias is evident for the studies that analyzed DE, funnel plot (see Fig. 3), and Egger's test 0.75, nor for those that analyzed the DTF Egger's test 0.73. (Additional file 1: Fig. S1).

### Heterogeneity of studies

Nineteen studies were included in the meta-analysis [1, 2, 15, 17–21, 25, 27-34, 36, 37], and seven studies were excluded from the meta-analysis. After all, their definition of success did not include the ability to maintain spontaneous breathing for 48 h after extubation, because they used other ultrasound measurements and because they did not present accurate data for quantitative analysis.

**Fig. 4:**
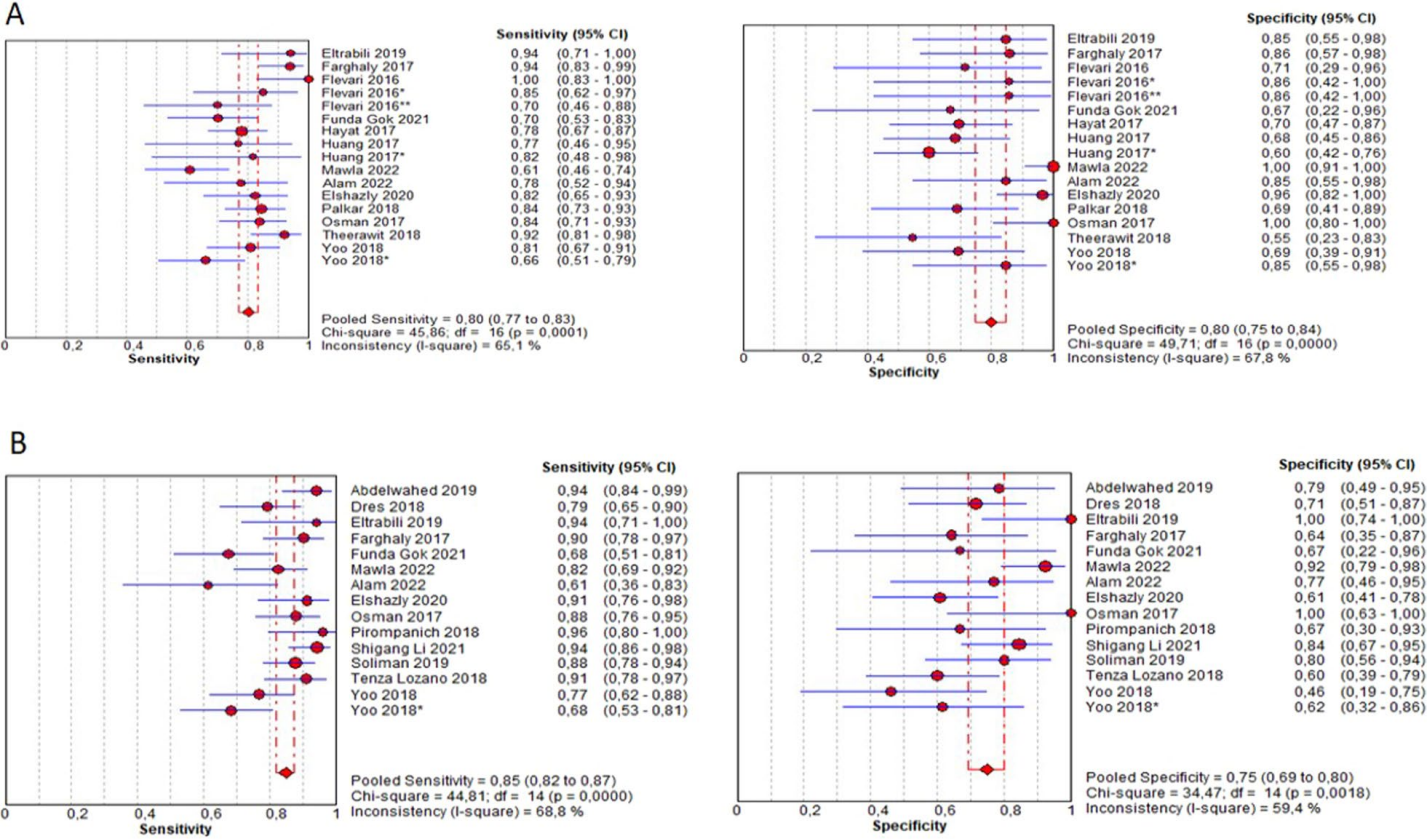
Forest plot of sensitivity and specificity (**A**) Diaphragmatic excursion. **B** Diaphragmatic thickening fraction. Flevari DE left > 10 mm*, **DE right < 10 mm; Huang*DE right; Yoo*DE ≥ 1.4 cm, DTF > 30%

**Fig. 5:**
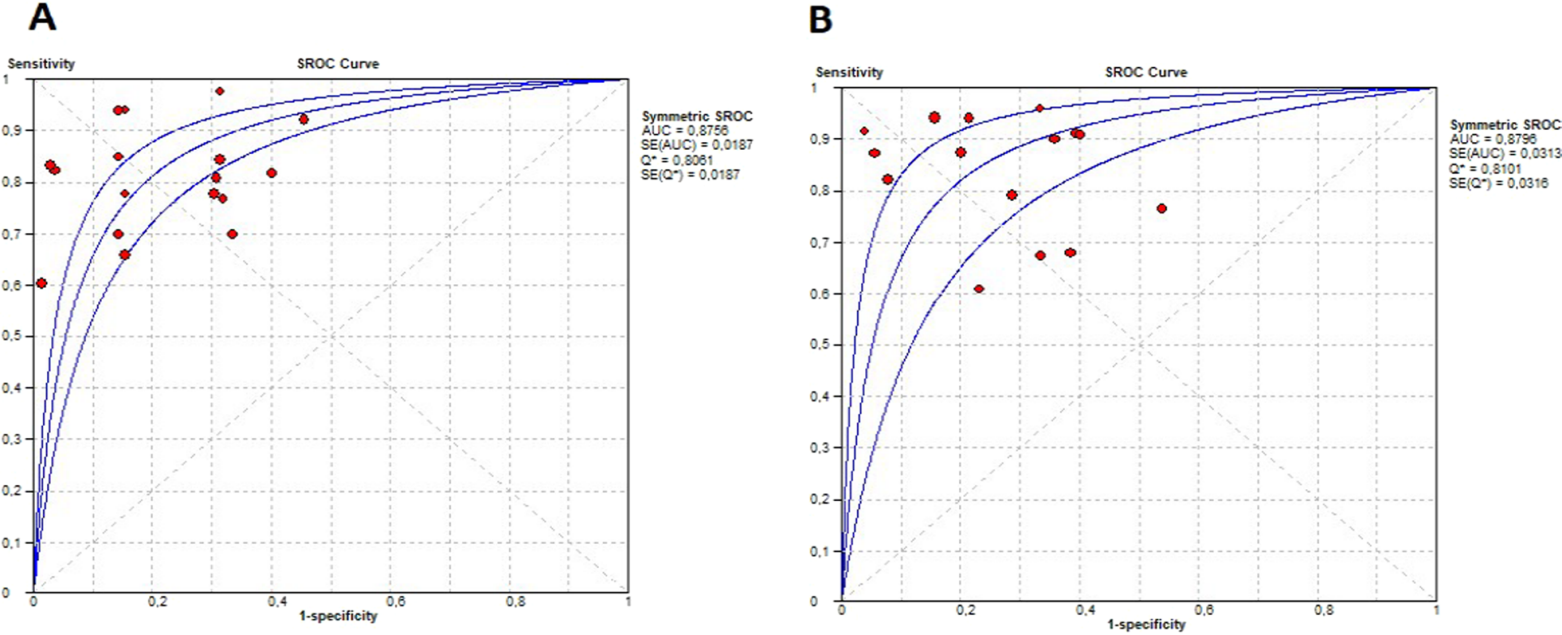
SROC curves of sensitivity and specificity (**A**) diaphragmatic excursion (**B**) diaphragmatic thickening fraction

Figure 4 presents the forest plot of sensitivity 0.80 (95% CI 0.77–0.83) and specificity 0.80 (95% CI 0.75–0.84) for DE and sensitivity 0.85 (95% CI 0.82–0.87) and specificity 0.75 (95% CI 0.69–0.80) for the DTF; Fig. 5 shows the SROC curve illustrating the summary point and the estimation of the sensitivity and specificity of each of the studies; also, the prediction contours with 95% CI and for DE AUSROC 0.87 and DTF AUSROC 0.87. The likelihood ratios obtained in the bivariate analysis for DE were L.R. (+) 4.64 (95% CI 4.19–5.0) L.R. (-) 0.21 (95% CI − 0.08–0.5) and for DTF L.R. (+) 3.5 (95% CI 3.19–3.84) LR (−) 0.18 (95% CI − 0.17–0.54) (see Additional file 1: Table S2).

Additional file 1: Fig. S2 shows the forest plot for the diagnostic odds ratio (DOR) for DE 17.1 (95% CI 10.2–28.6) and for DTF 17.2 (95% CI 9.16–32.3). Heterogeneity was evidenced in the sensitivity and specificity for DE (I^2^ 65.1% Chi-square 45.86 P 0.001; I^2^ 67.8% Chi-square 49.7 P 0.001, respectively) and for DTF (I^2^ 68.8% Chi-square 44.8 P 0.001; I^2^ 59.4% Chi-square 34.4 P 0.001); the threshold effect measure was evaluated by Spearman correlation, obtaining a value of 0.125 (P 0.63) for DE and − 0.198 (P 0.47) for DTF.

**Fig. 6:**
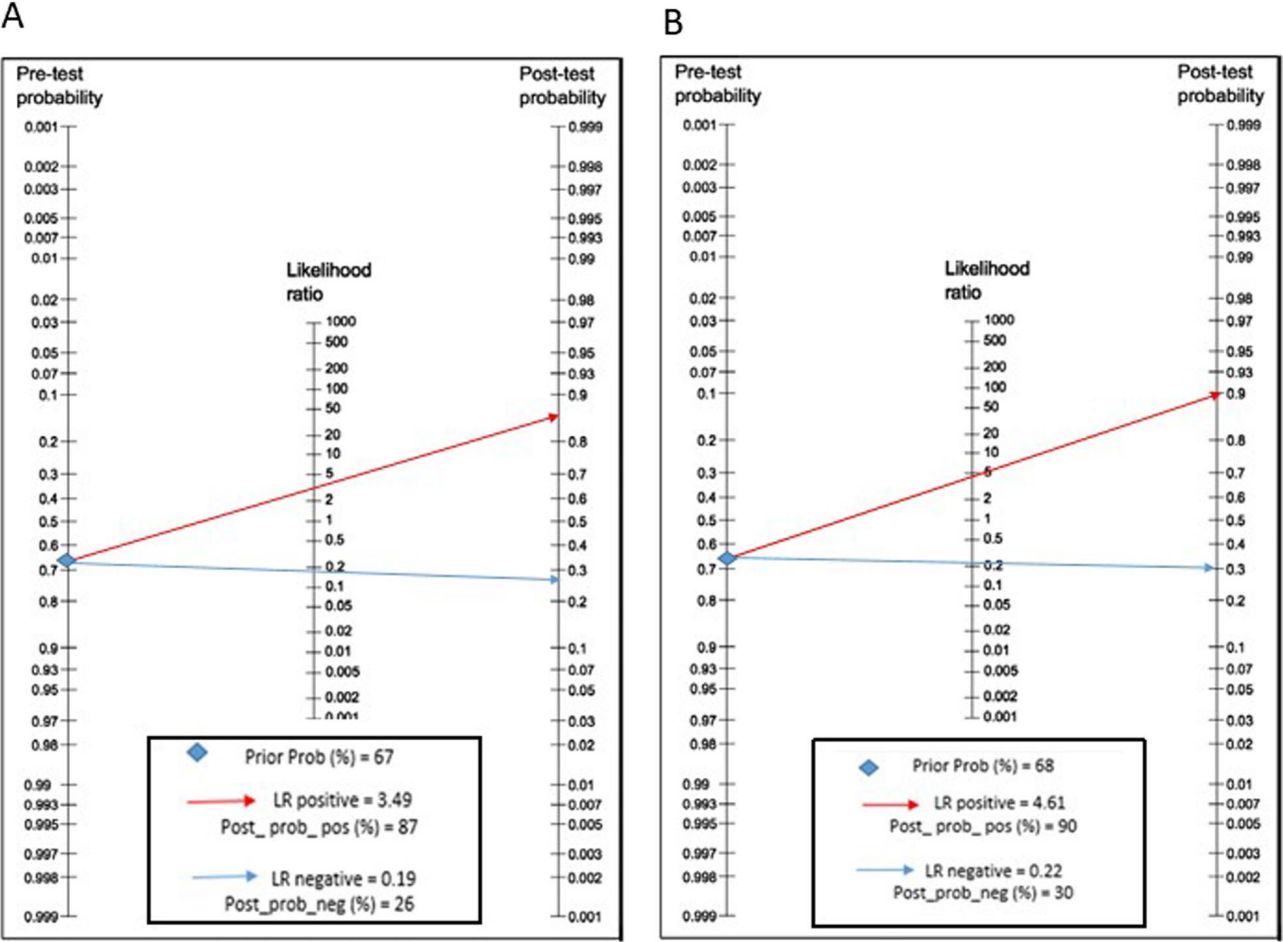
Fagan's nomogram for diaphragmatic excursion (**A**) and diaphragmatic thickening fraction (**B**)

A Fagan nomogram was constructed to illustrate diaphragm ultrasonography's pre- and post-test probability of predicting extubation success (Fig. 6). The pretest probability of ultrasonography predicting successful extubation was 67% for DE and 68% for positive DTF (above the cutoff point); the post-test probability for DE and DTF was 87% and 90%, respectively. The post-test probability of successful extubation for negative DE (below the cutoff point) was reduced to 26%, and for thickening, the DTF was reduced to 30%, respectively.

### Subgroup analysis and meta-regression

After eliminating studies with outlier DTF cutoff values [15, 20, 37], sensitivity increased to 0.86 (CI 0.83–0.89 I^2^ 68.5% Chi^2^ 34.9 P 0.002) with no changes in heterogeneity; specificity increased to 0.78 (0.72–0.83 I^2^ 56.9% Chi^2^ 25.5 P 0.01), with decreasing heterogeneity, and AUSROC increased to 0.90; continuing with the manual analysis by subgroups, studies with atypical values of sensitivity [15, 21] and specificity [32] for the DE were eliminated, showing an increase in sensitivity to 0.83 (95% CI 0.79–0.86) with a decrease in heterogeneity (I^2^ 43.9%, Chi^2^ 23.1 P 0.03); and decreased specificity to 0.77 (95% CI 0.71–0.83); likewise, decreased heterogeneity (I^2^ 52.9%, chi^2^ 27.5 P 0.01); the AUSROC increased to 0.88 (see Additional file 1: Fig. S3).

The exclusion of studies that presented a high risk of applicability according to the QUADAS 2 evaluation [15, 18] was performed, showing an increase in sensitivity to 0.87 (I^2^ 66% Chi^2^ 32.4 P 0.006) and specificity to 0.77 (I^2^ 60% Chi^2^ 27.7 P 0.003) without significant changes in the heterogeneity for DTF; concerning DE, there is an increase in sensitivity to 0.82 (I^2^ 60% chi^2^ 40 P 0.003) with no change in heterogeneity, no change in specificity for diaphragmatic excursion; the AUSROC was modified to 0.88 for a diaphragmatic excursion and 0.89 for DTF (see Additional file 1: Fig. S4).

A subgroup analysis was performed to evaluate whether the cause for which mechanical ventilation was required affects the diagnostic performance of ultrasound, eliminating studies where the main cause was not respiratory; there was no increase in sensitivity and specificity for DE 0.80 and 0.79, respectively, and AUSROC 0.86; similarly, there was no increase in sensitivity and specificity for thickening fraction 0.85 and 0.73, respectively, and AUSROC 0.86 (see Additional file 1: Fig. S5).

Finally, a subgroup analysis was performed for studies using PS and T-tube during the spontaneous breathing trial, with no evidence of statistically significant changes in sensitivity and specificity for DE and DTF (see Additional file 1: Fig. S6).

The meta regression analysis was performed, exploring possible sources of heterogeneity such as age, the prevalence of success, and patient position during the index test (diaphragmatic ultrasound); it was evidenced that patient position was a cause of heterogeneity for the diagnostic accuracy of the diaphragmatic thickening fraction in the studies included in the meta-analysis (coefficient of − 1,99 P = 0.012 DOR 0.14 CI 95% 0.03–0.6) (see Additional file 1: Table S3); no statistically significant differences were found between the age of patients who successfully weaned from mechanical ventilation and those who failed; likewise, there was no evidence that the prevalence of success was a source of heterogeneity.

## Discussion

The results of this study suggest adequate accuracy of diaphragmatic ultrasound in predicting weaning success; the combined sensitivity and specificity of DE and the AUSROC were 0.85, 0.75, 0.87, respectively, and for DTF 0.80, 0.80, 0.87. Our data show a satisfactory diagnostic accuracy for predicting extubation success. It is relevant to report that there was heterogeneity in the sensitivity and specificity of the studies included in the meta-analysis; likewise, several studies presented significant methodological weaknesses, two studies with a high risk of applicability in patient selection, and several studies with unclear risk of bias in patient selection, flow and timing.

During the last few years, some systematic reviews and meta-analyses have been published on the usefulness of diaphragmatic ultrasound in predicting the success or failure of weaning in patients undergoing mechanical ventilation [38–41]. The results of our study are consistent with most of the previously mentioned published studies. Li et al. evidenced in their study a satisfactory diagnostic accuracy in predicting the outcome of extubation; Llamas Alvarez concluded that DTF is by itself a modest predictor of weaning outcome; Garcia Sanchez et al. concluded that ultrasound dysfunction of the diaphragm is associated with an increased risk of extubation failure; Le Neindre et al. demonstrate that low values of diaphragmatic excursion and diaphragmatic thickening fraction predict the risk of extubation failure with moderate to high specificity.

This research included five new studies compared to the previous meta-analysis [41] that mainly analyzed DE and DTF, which allowed the number of subjects studied to be increased to 1204; likewise, an exhaustive subgroup analysis was performed to find sources of heterogeneity in the sensitivity and specificity of diaphragmatic excursion and diaphragmatic thickening fraction that could affect the ability of diaphragmatic ultrasound to predict extubation success, finding mainly factors such as cutoff values or atypical thresholds for each of the measurements and the risk of applicability found in the quality assessment of the studies; likewise, a bivariate meta-regression analysis was performed, finding the patient's position at the time of the test as the primary source of heterogeneity.

The high sensitivity values reported in the present study indicate that patients with values above approximately 29% for DTF and > 1 cm for DE have a high probability of successful extubation; however, it is essential to mention that weaning success may be influenced by additional factors such as nutritional status, respiratory and cardiovascular integrity and psychological conditions to mention a few [42].

We can evidence in the limitations of this study; the possible biases that may contain each of the studies included in the meta-analysis as randomized trials were not included; the absence of a common reference value for diaphragmatic thickening and excursion fraction may introduce biases that cause measurement imprecision; sex was not considered in the analysis by subgroups, nor the time on mechanical ventilation before the spontaneous breathing trial and ultrasound measurement; and these could influence the ultrasound result.

In contrast to the findings of this meta-analysis, the two studies mentioned here found no association between values below the cutoff point of diaphragmatic excursion and thickening fraction measured with ultrasound and the outcome of weaning from mechanical ventilation [24, 26]. Mariani et al. defined extubation failure as the need for intubation within 72 h after extubation, and Vivier et al. defined extubation failure as the need for intubation or death seven days after extubation, which differs from our investigation since the studies included in this meta-analysis evaluated extubation success 48 h after mechanical ventilator weaning.

Slight variations in the measurement between observers may affect the measurement result and cause heterogeneity; it is undoubtedly an observer-dependent technique; despite this, several studies have concluded that diaphragmatic ultrasound measurements are reproducible.

The results of this study have implications for clinical practice, showing that diaphragmatic ultrasound is a technique that can be used in the intensive care unit during the spontaneous breathing trial to contribute to objectively predict the success of weaning from mechanical ventilation; it is a portable, fast, noninvasive, simple and safe technique that does not emit any ionizing radiation that affects health-care personnel; However, given the high heterogeneity found, which is frequent in meta-analyses of diagnostic tests, the results of the pooled measurements should be interpreted with caution, especially in the different subgroups of critically ill patients, in order to achieve a personalized determination of the optimal result.

Therefore, access to diaphragmatic ultrasound in the intensive care unit should be generalized, and priority should be given to achieving its universal use, especially in routine respiratory monitoring, to guide the management of patients undergoing mechanical ventilation.

## Conclusions

The results of this systematic review and meta-analysis show that measurement of diaphragmatic excursion and diaphragmatic thickening fraction predict the probability of successful weaning from mechanical ventilation with satisfactory diagnostic accuracy; however, significant heterogeneity was evident in the different included studies. Studies of high methodological quality in specific subgroups in intensive care unit patients are needed to evaluate the role of diaphragmatic ultrasound as a predictor of weaning from mechanical ventilation.

## Supplementary Information

**Additional file 1.** Search strategy, supplemental Tables, and supplemental Figures.

## Data Availability

The datasets used and/or analyzed during the current study are available from the corresponding author on reasonable request.

## Abbreviations

ICU: Intensive care unit
SBT: Spontaneous breathing trial
QUADAS-2: Quality Assessment of Diagnostic Accuracy Studies-2
SROC: Summary receiver operator characteristic curve
AUSROC: Area under the summary receiver operator characteristic curve
DE: Diaphragmatic excursion
DTF: Diaphragmatic thickening fraction
PS: Pressure support
DRSBI: Diaphragmatic rapid shallow breathing index
PEEP: Positive end-expiratory pressure
