# Supplementary material for: Effectiveness of diaphragmatic ultrasound as a predictor of successful weaning from mechanical ventilation: a systematic review and meta-analysis
Source: Crit Care. 2023 May 5;27:174. doi: 10.1186/s13054-023-04430-9 (PMC10161591; doi:10.1186/s13054-023-04430-9)
Supplement: Supplementary file 1 — Additional file 1. Search strategy, supplemental Tables, and supplemental Figures. [file 13054_2023_4430_MOESM1_ESM.docx]

**ADDITIONAL FILES**

**Article search strategy**

(("Ultrasonography"[Mesh]) AND "Diaphragm"[Mesh]) AND "Intensive Care Units"[Mesh]

(((("diagnostic imaging" [Subheading])) OR "Ultrasonography"[Mesh]) AND "Ventilator Weaning"[Mesh]) AND "Airway Extubation"[Mesh]

((((("Ultrasonography"[Mesh]) OR "diagnostic imaging" [Subheading]) AND "Diaphragm"[Mesh]) AND "Intensive Care Units"[Mesh]) AND "Ventilator Weaning"[Mesh]) AND "Airway Extubation"[Mesh]

("diagnostic imaging"[MeSH Subheading] OR ("diagnostic"[All Fields] AND "imaging"[All Fields]) OR "diagnostic imaging"[All Fields] OR "ultrasound"[All Fields] OR "ultrasonography"[MeSH Terms] OR "ultrasonography"[All Fields] OR "ultrasonics"[MeSH Terms] OR "ultrasonics"[All Fields] OR "ultrasounds"[All Fields] OR "ultrasound s"[All Fields]) AND ("diaphragm"[MeSH Terms] OR "diaphragm"[All Fields] OR "contraceptive devices, female"[MeSH Terms] OR ("contraceptive"[All Fields] AND "devices"[All Fields] AND "female"[All Fields]) OR "female contraceptive devices"[All Fields] OR "diaphragms"[All Fields] OR "diaphragm s"[All Fields] OR "diaphragmal"[All Fields])

"diagnostic imaging"[Subheading] OR ("diagnostic"[All Fields] AND "imaging"[All Fields]) OR "diagnostic imaging"[All Fields] OR "ultrasound"[All Fields] OR "ultrasonography"[MeSH Terms] OR "ultrasonography"[All Fields] OR "ultrasonics"[MeSH Terms] OR "ultrasonics"[All Fields] OR "ultrasounds"[All Fields] OR "ultrasound's"[All Fields]

"diaphragm"[MeSH Terms] OR "diaphragm"[All Fields] OR "contraceptive devices, female"[MeSH Terms] OR ("contraceptive"[All Fields] AND "devices"[All Fields] AND "female"[All Fields]) OR "female contraceptive devices"[All Fields] OR "diaphragms"[All Fields] OR "diaphragm's"[All Fields] OR "diaphragmal"[All Fields]

(((("Ultrasonography"[Mesh]) OR "Diagnostic Imaging"[Mesh]) AND "Intensive Care Units"[Mesh]) AND "Ventilator Weaning"[Mesh]) NOT "Pediatrics"[Mesh]

("diaphragm"[MeSH Terms] OR "diaphragm"[All Fields] OR "diaphragmatic"[All Fields]) AND ("diagnostic imaging"[MeSH Subheading] OR ("diagnostic"[All Fields] AND "imaging"[All Fields]) OR "diagnostic imaging"[All Fields] OR "ultrasonography"[All Fields] OR "ultrasonography"[MeSH Terms] OR "ultrasonographies"[All Fields]) AND ("critical care"[MeSH Terms] OR ("critical"[All Fields] AND "care"[All Fields]) OR "critical care"[All Fields] OR ("intensive"[All Fields] AND "care"[All Fields]) OR "intensive care"[All Fields])

("diaphragm"[MeSH Terms] OR "diaphragm"[All Fields] OR "diaphragmatic"[All Fields]) AND ("diagnostic imaging"[MeSH Subheading] OR ("diagnostic"[All Fields] AND "imaging"[All Fields]) OR "diagnostic imaging"[All Fields] OR "ultrasonography"[All Fields] OR "ultrasonography"[MeSH Terms] OR "ultrasonographies"[All Fields]) AND ("critical care"[MeSH Terms] OR ("critical"[All Fields] AND "care"[All Fields]) OR "critical care"[All Fields] OR ("intensive"[All Fields] AND "care"[All Fields]) OR "intensive care"[All Fields]) AND ("success"[All Fields] OR "successes"[All Fields] OR "successful"[All Fields]) AND ("weaning"[MeSH Terms] OR "weaning"[All Fields] OR "weaned"[All Fields] OR "weanings"[All Fields] OR "weans"[All Fields])

(("diaphragm"[MeSH Terms] OR "diaphragm"[All Fields] OR "diaphragmatic"[All Fields]) AND ("diagnostic imaging"[MeSH Subheading] OR ("diagnostic"[All Fields] AND "imaging"[All Fields]) OR "diagnostic imaging"[All Fields] OR "ultrasonography"[All Fields] OR "ultrasonography"[MeSH Terms] OR "ultrasonographies"[All Fields]) AND ("critical care"[MeSH Terms] OR ("critical"[All Fields] AND "care"[All Fields]) OR "critical care"[All Fields] OR ("intensive"[All Fields] AND "care"[All Fields]) OR "intensive care"[All Fields]) AND ("success"[All Fields] OR "successes"[All Fields] OR "successful"[All Fields]) AND ("weaning"[MeSH Terms] OR "weaning"[All Fields] OR "weaned"[All Fields] OR "weanings"[All Fields] OR "weans"[All Fields])) NOT ("paediatrics"[All Fields] OR "pediatrics"[MeSH Terms] OR "pediatrics"[All Fields] OR "paediatric"[All Fields] OR "pediatric"[All Fields])

(("diaphragm"[MeSH Terms] OR "diaphragm"[All Fields] OR "diaphragmatic"[All Fields]) AND ("diagnostic imaging"[MeSH Subheading] OR ("diagnostic"[All Fields] AND "imaging"[All Fields]) OR "diagnostic imaging"[All Fields] OR "ultrasound"[All Fields] OR "ultrasonography"[MeSH Terms] OR "ultrasonography"[All Fields] OR "ultrasonics"[MeSH Terms] OR "ultrasonics"[All Fields] OR "ultrasounds"[All Fields] OR "ultrasound s"[All Fields]) AND ("critical care"[MeSH Terms] OR ("critical"[All Fields] AND "care"[All Fields]) OR "critical care"[All Fields] OR ("intensive"[All Fields] AND "care"[All Fields]) OR "intensive care"[All Fields]) AND ("success"[All Fields] OR "successes"[All Fields] OR "successful"[All Fields]) AND ("weaning"[MeSH Terms] OR "weaning"[All Fields] OR "weaned"[All Fields] OR "weanings"[All Fields] OR "weans"[All Fields])) NOT ("infant, newborn"[MeSH Terms] OR ("infant"[All Fields] AND "newborn"[All Fields]) OR "newborn infant"[All Fields] OR "neonatal"[All Fields] OR "neonate"[All Fields] OR "neonates"[All Fields] OR "neonatality"[All Fields] OR "neonatals"[All Fields] OR "neonate s"[All Fields])

**Question**: Should diaphragmatic ultrasound be used to diagnose the success of weaning from mechanical ventilation in the intensive care unit?

**diaphragmatic excursion thickening fraction**

| \| Sensitivity \| 0.80 (95% CI: 0.77 to 0.83) \| \| --- \| --- \| \| Specificity \| 0.80 (95% CI: 0.75 to 0.84) \| |  | \| Sensitivity \| 0.85 (95% CI: 0.82 to 0.87) \| \| --- \| --- \| \| Specificity \| 0.75 (95% CI: 0.69 to 0.80) \| |  |
| --- | --- | --- | --- | --- | --- | --- | --- | --- | --- | --- | --- |

| **Outcome** | **№ of studies (№ of patients)** | **Study design** | **Factors that may decrease certainty of evidence** | | | | | **Test accuracy CoE** | **Importance** |
| --- | --- | --- | --- | --- | --- | --- | --- | --- | --- |
|  |  |  | **Risk of bias** | **Indirectness** | **Inconsistency** | **Imprecision** | **Publication bias** |  |  |
| **True positives** (patients with weaning success) | 19 studies 1204 patients | cross-sectional (cohort type accuracy study)  observational studies | serious^a^ | not serious | serious^b^ | not serious | all plausible residual confounding would reduce the demonstrated effect | ⨁⨁⨁◯ Moderate | IMPORTANT |
| **False negatives** (patients incorrectly classified as not having weaning success) |  |  |  |  |  |  |  |  | IMPORTANT |
| **True negatives** (patients without weaning success) | 19 studies 1204 patients | cross-sectional (cohort type accuracy study)  observational studies | Serious^a^ | not serious | Serious ^b^ | not serious | all plausible residual confounding would reduce the demonstrated effect | ⨁⨁⨁◯ Moderate | IMPORTANT |
| **False positives** (patients incorrectly classified as having weaning success) |  |  |  |  |  |  |  |  | IMPORTANT |
| **Inconclusive** | 0 studies 0 patients | - | - | - | - | - | - | - |  |
| **Complications** | 0 studies patients |  |  |  |  |  |  | - |  |

#### Explanations

a. Biases may occur in some studies due to the methodological design “no serious limitations”.

b. A significant degree of heterogeneity is evident in the included studies as measured with I^2^ and Q cochrane.

Table 1. Additional summary table of the results of the evaluation of the certainty of the articles included in the meta-analysis using the Grading of Recommendations Assessment, Development, and Evaluation methodology (GRADE).

|  | **Estimate** | **[ 95% Confidence Interval]** | |
| --- | --- | --- | --- |
| **Diaphragm Excursión** | | | |
| Sensitivity | 0.80 | 0.77 | 0.83 |
| Specificity | 0.80 | 0.75 | 0.84 |
| DOR | 17.1 | 10.2 | 28.6 |
| LR+ | 4.64 | 4.19 | 5.0 |
| LR- | 0.21 | -0.08 | 0.5 |
| **Diaphragm Thickening Fraction** | | | |
| Sensitivity | 0.85 | 0.82 | 0.87 |
| Specificity | 0.75 | 0.69 | 0.80 |
| DOR | 17.2 | 9.16 | 32.3 |
| LR+ | 3.5 | 3.19 | 3.84 |
| LR- | 0.18 | -0.17 | 0.54 |

Table 2 Additional Results of sensitivity, specificity, L.R. (+), L.R. (-) DOR measurements for diaphragmatic excursion and diaphragmatic thickening fraction.


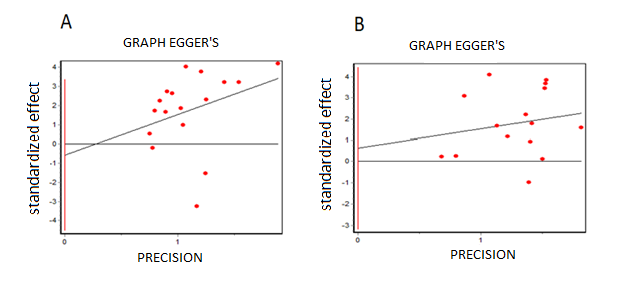


Figure 1 Additional Egger's statistic results for diaphragmatic excursion (A) and diaphragmatic thickening fraction (B).

**
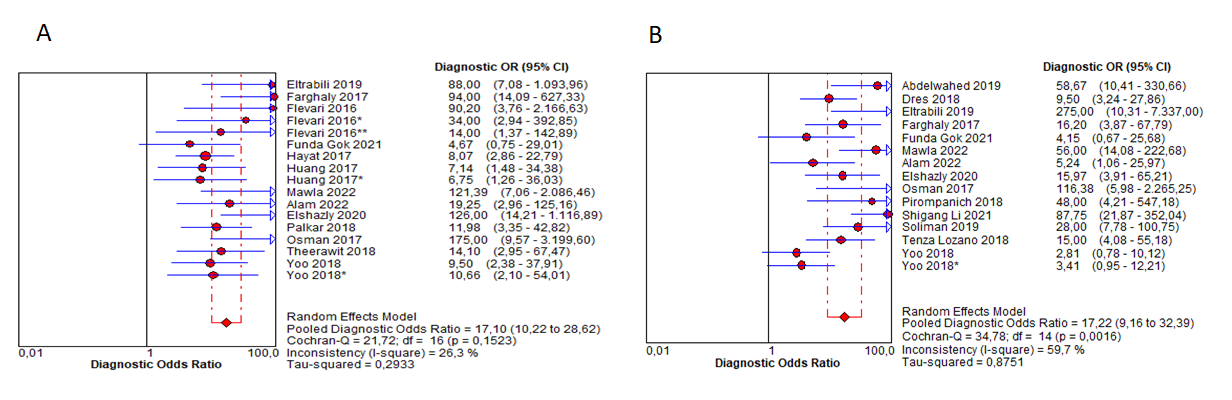
**

Figure 2 Additional DOR diaphragmatic excursion (A) DOR diaphragmatic thickening fraction (B).

**
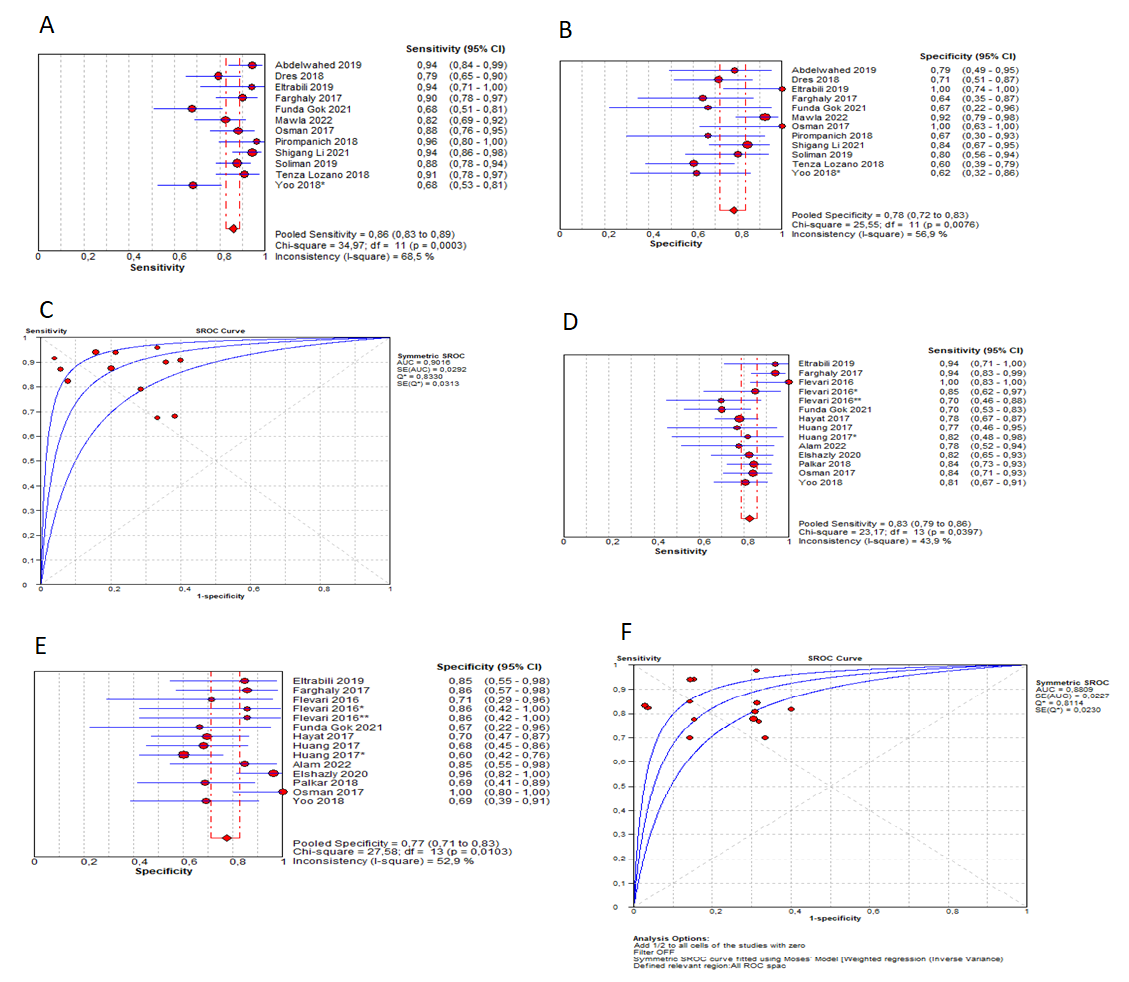
**

Figure 3 Additional sensitivity, specificity, and SROC (A, B, C) after removing the outlier for diaphragmatic thickening fraction and sensitivity and specificity and SROC (D, E, F) after removing outlier the sensitivity and specificity for a diaphragmatic excursion.

**
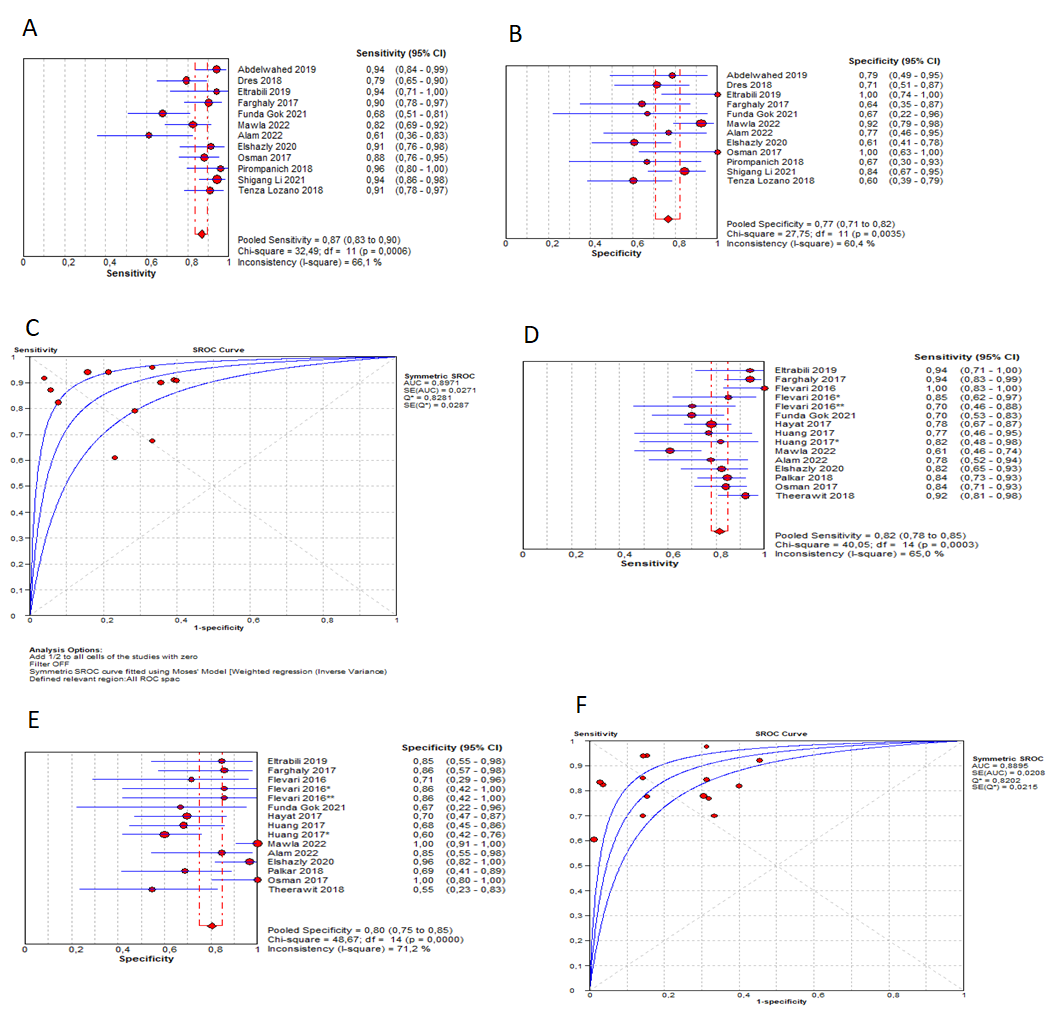
**

Figure 4 Additional sensitivity and specificity and SROC after eliminating studies with a high risk of applicability according to QUADAS 2 for diaphragm thickening fraction (A, B, C) and diaphragmatic excursion (D, E, F).


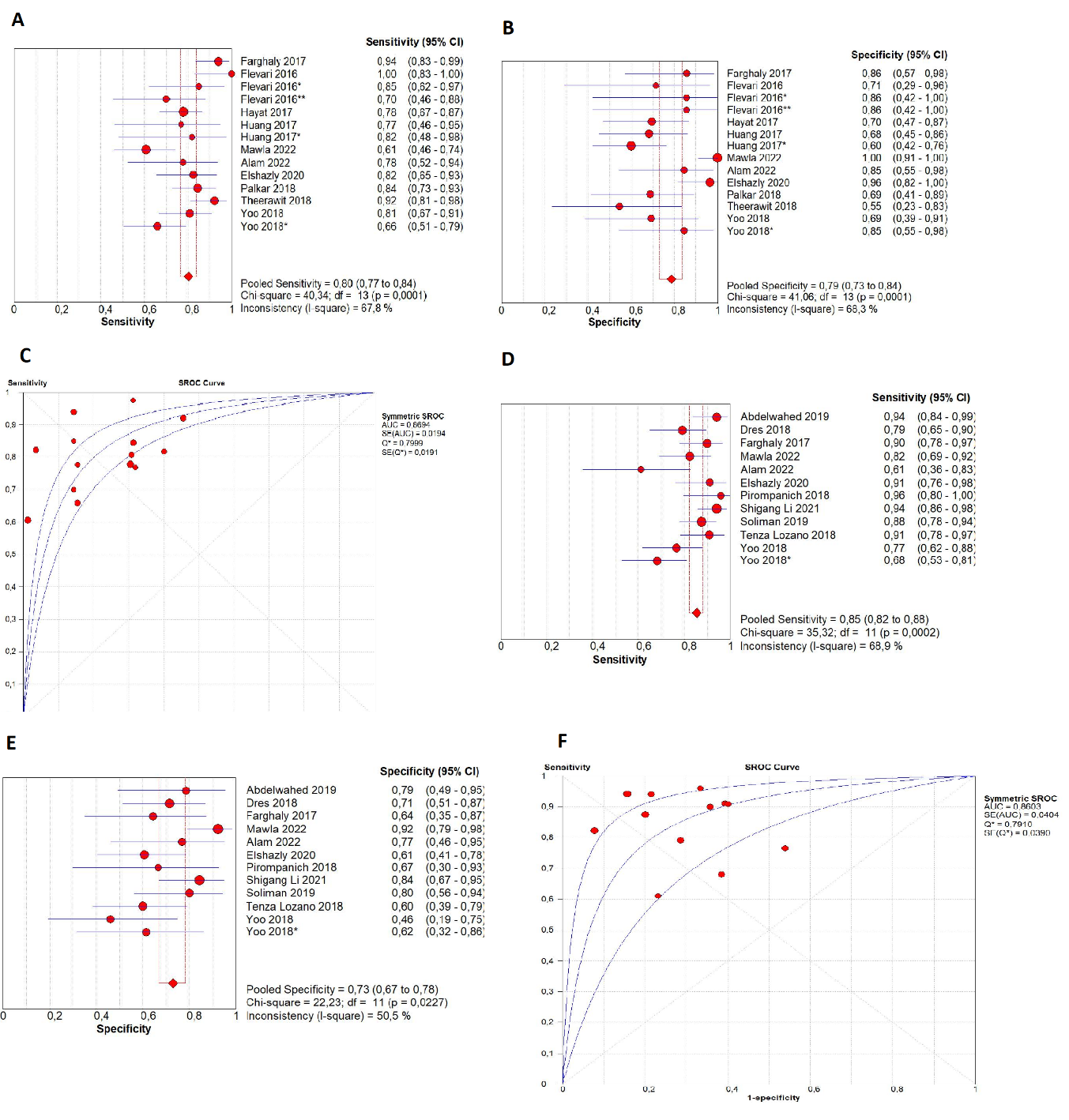


Figure 5 Additional sensitivity and specificity and SROC after eliminating studies whose cause of mechanical ventilation was not respiratory for diaphragmatic excursion (A, B, C) diaphragm thickening fraction (D, E, F).

**
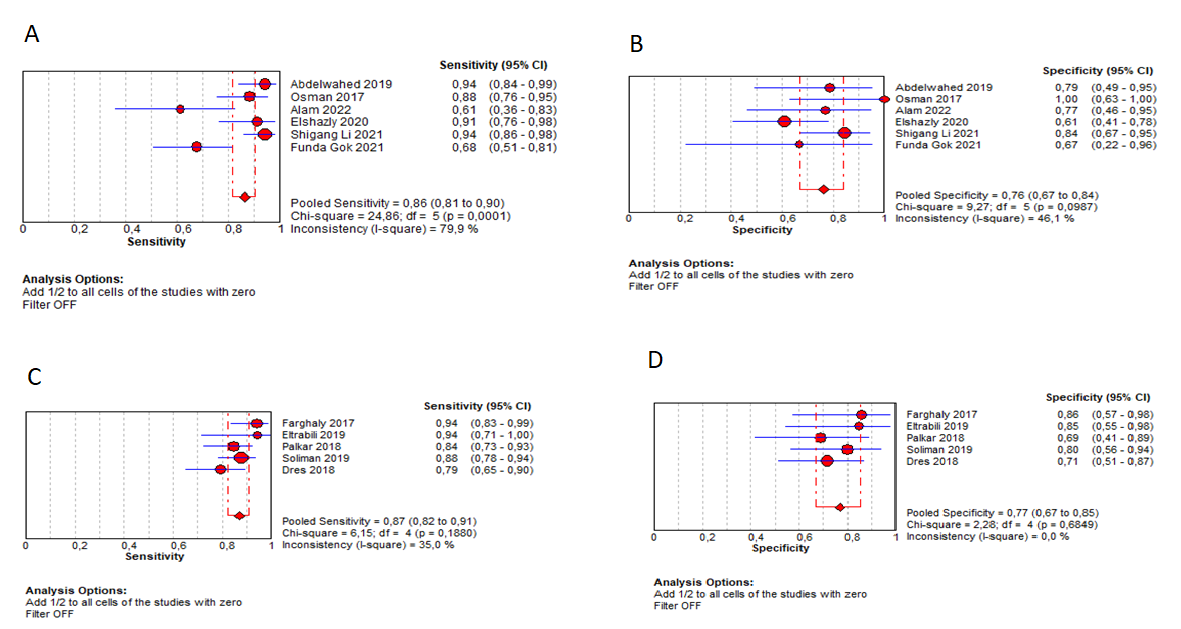
**

Figure 6 Additional sensitivity and specificity for predicting weaning success of studies using T-tube alone (A, B) and PSV (C, D).

| **META-REGRESSION ANALYSIS** | | | | | |
| --- | --- | --- | --- | --- | --- |
| **TEST INDEX** | **COVARIATE** | **COEFFICIENT** | **P VALUE** | **R DOR** | **IC 95%** |
| **DE** | **PREVALENCE OF SUCCESSFUL WEANING** | 0,223 | 0,69 | 1,25 | 0,38-4,06 |
|  | **HOMOGENEITY VS. HETEROGENEITY OF AGE** | 0,429 | 0,53 | 1,54 | 0,36-6,55 |
|  | **PATIENT POSITIONING DURING THE TEST** | -0,033 | 0,95 | 0,97 | 0,29-3,21 |
| **DTF** | **PREVALENCE OF SUCCESSFUL WEANING** | 0,046 | 0,95 | 1,05 | 0,21-5,17 |
|  | **HOMOGENEITY VS. HETEROGENEITY OF AGE** | -1,22 | 0,23 | 0,29 | 0,03-2,5 |
|  | **PATIENT POSITIONING DURING THE TEST** | -1,997 | 0,012 | 0,14 | 0,03.0,6 |

Table 3. Additional results of meta- regression.
